# Genetic or Psychogenic? A Case Study of “Folie à Quatre” Including Twins

**DOI:** 10.1155/2015/983212

**Published:** 2015-06-25

**Authors:** Tohru Ohnuma, Heii Arai

**Affiliations:** Juntendo University Schizophrenia Projects (JUSP), Department of Psychiatry, Faculty of Medicine, Juntendo University, Tokyo, Japan

## Abstract

Shared psychotic disorder, characterized by shared delusion among two or more subjects (termed “Folie à deux,” “trois,” etc.), is often associated with strong religious beliefs or social isolation, factors creating strong psychological sympathy. Recently, we treated a rare familial case of “Folie à quatre” in central Tokyo without such influences. The proband was a schizophrenia patient and younger brother within monozygotic twins. Positive symptoms were “transmitted” to remaining family members, his elder brother, mother, and father father, in a relatively short period of three months. Although the pathophysiology of these positive symptoms (delusions and hallucinations) remains unclear, the transmission pattern suggests the primacy of social and environmental factors (and/or their interaction), while genetics appeared less influential in this “Folie à famille.” Although undiagnosed psychoses in the whole family cannot be excluded, they did not share the other negative schizophrenia symptoms of the proband. A strong familial connection appeared to be the most important factor for the common delusion and hallucination.

## 1. Introduction

Shared psychotic disorder is usually “Folie à deux” (delusions shared by two individuals) [1, 2] or more rarely “Folie à trois” [3], while “Folie à quatre” and delusions shared by more than four individuals are extremely rare. Indeed, only a few cases of “Folie à quatre” [4–6] and “Folie à cinq” [7, 8] have been reported. In recent years, we are aware of only one such case of “Folie à quatre” in Japan [9]. Recently, we treated a familial case of “Folie à quatre” involving a proband who was a younger brother in a set of monozygotic twins. His delusions and visual hallucinations were transmitted to his older twin brother, mother, and father in a relatively short period of three months after onset. This rare case highlights the importance of shared environmental factors (strong familial bonds) as discussed in a recent review [10]. However, this case did not involve strong religiosity or a closed environment, which are frequent factors in shared psychotic disorder.

## 2. Case Presentation

The family consists of the then 30-year-old male proband (A), his 30-year-old elder monozygotic twin brother (B), 56-year-old mother (C), and 60-year-old father (D). At the time of presentation, B was working in an office usually burdened with overtime on weekdays, C was a housewife, and D was also working in an office doing regular shifts. The family had lived together for 30 years in a detached house in the 23rd ward of central Tokyo, Japan. The family did not seek further treatment and could not be contacted following the initial study. Informed consent was not requested prior to loss of contact. Nonetheless, anonymity is assured by this description, and the case is of sufficient interest and importance to justify publication.

Present illness: the proband was born as the younger male of monozygotic twins. No features of developmental disorders or mental retardation were recognized during elementary or junior high school. After graduating high school, he worked as a truck driver until the age of 26 years. At 26, he was smoking 2-3 cigars per day, and the odor was addressed by his seniors in the office (“I get a strong smell of something burning from you”). The smell became gradually worse and he resigned; since then, he has been in social withdrawal. He has also exhibited typical negative symptoms of schizophrenia, such as emotional withdrawal and motor retardation, over the past 4 years. Three months after resigning, he felt something “like insects” moving in his body and something like “jelly” hanging from his throat all the way down to his stomach. Later, he had an internal medicine examination, which revealed nothing. This delusion was soon accompanied by hallucinations that “the jelly-like substance or amoeboid materials come out from my mouth, and people around me react to those materials” and “the materials are accumulating in my body; thus, my muscles and body fat are melting, please conduct a biopsy.” He and his family visited numerous hospitals, but examinations showed no abnormalities. Later, in order to get rid of the amoeboid materials coming out of his mouth, he consumed five boxes of Kleenex.

After about one month, his mother, who spent the most time with him, also began to see the amoeboid materials. “First, I thought what nonsense he said, but later I also began to see the amoeboid materials coming out with twinkle-like ectoplasm. Surrounding people drinking in the neighboring bar and even dogs were apparently affected by it, thus they are coughing and drooling.” The elder brother, who spent the least amount of time with A, also began seeing the ectoplasm coming out from A's mouth starting about 2 months after the appearance of A's positive symptoms. Furthermore, B himself was affected by the material from A's mouth. He coughed at the “sight” of it and was sure that the materials were also noticed by other people riding the train with A. Finally, three months after onset of A's delusions, the father, who also spent less time with A than C (but more time than B), started to see the materials and felt that this affected other people in the presence of A.

The proband was seeing a psychiatrist with B and C, and A was also examined by doctors in other departments. We conducted counseling for A and heard about the transmission of symptoms to B and C. They also believed doctors in other departments could see the materials. We assumed “Folie à quatre” (including D, his father) based on these shared delusions and hallucinations. We could easily determine that only the proband had schizophrenia as (1) A showed the symptoms first and (2) only A exhibited social withdrawal caused by typical negative symptoms (he was poorly dressed with dirty cloths and dirty long hair). We carefully listened to his symptoms with sympathy and did not deny his experience. Unfortunately, he asked whether we could see the materials coming out of his mouth; when we told him that we could not, A said “I am tired of explaining it to doctors. I will only meet doctors who can see it” and he never returned.

## 3. Discussion

Here we presented a familial case of “Folie à quatre” in which the delusions and hallucinations of the proband (a 30-year-old schizophrenia patient) were eventually shared by the rest of the family, his monozygotic twin brother and parents, none of whom exhibited other signs consistent with schizophrenia such as negative symptoms. Moreover, this case did not involve overt extreme religiosity or prolonged isolation (the family lived in central Tokyo).

Only the proband (A) was diagnosed with schizophrenia according to the Diagnostic and Statistical Manual of Mental Disorders 5 (DSM-5) [11]. The concurrence of schizophrenia is ≈50% in the other monozygotic twin and ≈5% in parents of a schizophrenic patient [12]; however, neither the brother nor parents initially shared the delusions and hallucinations and they never presented with negative symptoms. Further, symptoms were not present for over 6 months. Thus, we could distinguish schizophrenia from “delusional symptoms in partner with delusional disorder” based on these features.

The etiology of transmission, whether by chance, genetic factors, environmental factors, and/or some interaction among them, is a matter of speculation. Although A also showed typical negative symptoms of schizophrenia, such as emotional withdrawal and motor retardation antecedent to delusion and hallucination, only the latter positive symptoms were transmitted. The mother spent the greatest amount of time with A and exhibited shared delusions and hallucinations faster than the equally related father and more closely related twin brother, both of whom spent less time with A on any given day. Thus, shared environmental factors appear more influential than genetics. Nonetheless, the monozygotic twin brother spent less time with the proband than the father (due to daytime work and overtime) but manifested the shared delusion faster than the father. This may be explained by the “genetic sympathy” of monozygotic twins as discussed in previous reviews [13, 14] rather than shared genetics per se. The father manifested such symptoms much later than the mother, again suggesting environmental over genetic influences. In addition, D would eventually be exposed to the most pervasive environmental influence as all other family members shared the delusion and hallucination for about one month before he too reported them. Thus, for establishing “Folie à famille,” extreme religiosity, isolation, and genetics do not appear necessary; rather a strong familial connection was the most important factor for transmission. A previous case published in Japanese described a case of “Folie à quatre” that did not include twins, a religious factor, or isolation (the family lived in a newly established suburb of Kyusyu, Japan) [9]. The present case is distinct from previous instances described in a case report [15] and review [16] on “Folie à deux” in monozygotic twins, as another family member with less genetic similarity (the mother) reported the delusion and hallucination first. Shared psychosis usually involves shared delusions, but the present monozygotic twin case and some previous cases also exhibited shared hallucinations (auditory or visual). Thus, genetic factors may contribute to shared hallucinations. While it is possible that the whole family had undiagnosed psychoses following independent trajectories, no other family members had a history of schizophrenia, none had negative symptoms, and the time lag in onset appeared more consistent with shared environment than the strength of the genetic relationship. Nonetheless, as the family could not be studied over a longer period, this possibility must be considered as a limitation in the present report.

In the operational diagnostic criteria in DSM-5 published last year, there is no longer a distinction between shared delusional disorder and delusional disorder. Thus, those sharing the delusion have delusion disorder if meeting the other criteria or “other specified schizophrenia spectra and other psychotic disorders” if not. Environmental factors are stronger influences on those sharing the delusion than on the proband (with endogenous psychosis). Such cases are usually treated by psychotherapy and isolation from the proband, but usually without antipsychotics. For those sharing the delusion, “Folie à deux” appears better interpreted as a fragmental psychotic symptom of dissociative disorder (F44) from the point of the aforementioned psychogenic factors.
